# Calcium Signaling and Gliotransmission in Normal vs. Reactive Astrocytes

**DOI:** 10.3389/fphar.2012.00139

**Published:** 2012-07-13

**Authors:** Cendra Agulhon, Min-Yu Sun, Thomas Murphy, Timothy Myers, Kelli Lauderdale, Todd A. Fiacco

**Affiliations:** ^1^UFR Biomédicale, CNRS UMR 8154, Université Paris DescartesParis, France; ^2^Department of Cell Biology and Neuroscience, Center for Glial-Neuronal Interactions, University of California RiversideRiverside, CA, USA; ^3^Program in Cellular, Molecular, and Developmental Biology, University of California RiversideRiverside, CA, USA

**Keywords:** glia, IP_3_R, microglia, neurological disorder, neurodegenerative disease, TNFα, G_q_ GPCR, inflammation

## Abstract

A prominent area of neuroscience research over the past 20 years has been the acute modulation of neuronal synaptic activity by Ca^2+^-dependent release of the transmitters ATP, D-serine, and glutamate (called gliotransmitters) by astrocytes. Although the physiological relevance of this mechanism is under debate, emerging evidence suggests that there are critical factors in addition to Ca^2+^ that are required for gliotransmitters to be released from astrocytes. Interestingly, these factors include activated microglia and the proinflammatory cytokine Tumor Necrosis Factor α (TNFα), chemotactic cytokine Stromal cell-Derived Factor-1α (SDF-1α), and inflammatory mediator prostaglandin E2 (PGE_2_). Of note, microglial activation and release of inflammatory molecules from activated microglia and reactive astrocytes can occur within minutes of a triggering stimulus. Therefore, activation of astrocytes by inflammatory molecules combined with Ca^2+^ elevations may lead to gliotransmitter release, and be an important step in the early sequence of events contributing to hyperexcitability, excitotoxicity, and neurodegeneration in the damaged or diseased brain. In this review, we will first examine evidence questioning Ca^2+^-dependent gliotransmitter release from astrocytes in healthy brain tissue, followed by a close examination of recent work suggesting that Ca^2+^-dependent gliotransmitter release occurs as an early event in the development of neurological disorders and neuroinflammatory and neurodegenerative diseases.

## Introduction

Gliotransmission – defined as the rapid and regulated exocytosis of gliotransmitters by astrocytes in an activity and G_q_ G-protein-coupled receptor (GPCR), Ca^2+^-dependent manner – has continued to be an exciting area of neuroscience research ever since its initial discovery in 1994 (Parpura et al., 1994). Because the tight regulation provided by Ca^2+^ elevations and release by quantal-like vesicular exocytosis are typical properties of a physiological process (Bezzi et al., 2001), astrocytes have been touted as active, not only supportive, partners in the control of rapid synaptic communication in the healthy brain (Haydon, 2001; Volterra and Meldolesi, 2005; Perea et al., 2009; Santello and Volterra, 2009).

However, studies using novel techniques to selectively evoke or block G_q_ GPCR-mediated Ca^2+^ elevations in astrocytes have failed to observe changes in neuronal excitatory synaptic activity indicative of gliotransmission (Fiacco et al., 2007; Petravicz et al., 2008; Agulhon et al., 2010). This has raised controversy as to whether gliotransmission actually occurs in healthy brain tissue. Emerging evidence is providing a possible explanation for this discrepancy: Ca^2+^ elevations alone are not sufficient for gliotransmitter release by astrocytes. Additional factors such as inflammatory molecules appear to be required. These molecules may not be present in adequate quantities in healthy brain tissue to elicit sufficient gliotransmitter release to affect synaptic transmission. However, accumulating evidence suggests that in the early steps of inflammatory processes and in the diseased or damaged brain, activated microglia, and astrocytes engage in a partnership that transforms astrocytes, within seconds to minutes, into competent gliotransmitter releasing cells. As astrocytic gliotransmitters include ATP, d-serine, and glutamate, reactive astrocytes could increase neuronal excitability and contribute to excitotoxicity, synaptic damage, and pathogenesis of disease. Therefore, inflammatory transduction pathways that regulate Ca^2+^-dependent release of gliotransmitters from astrocytes are potential targets for therapy of neurological disorders as well as neuroinflammatory and neurodegenerative disease.

In this review, we will first examine evidence questioning Ca^2+^-dependent gliotransmitter release by astrocytes in the healthy brain, including the mechanisms driving astrocyte Ca^2+^ elevations, differences in basal (resting) Ca^2+^ vs. activity-induced Ca^2+^ elevations, and the temporal relationship between activation of postsynaptic receptors and astrocyte Ca^2+^ elevations. We will then review evidence suggesting that activated microglia and release of inflammatory mediators transform astrocytes into cells capable of releasing gliotransmitters in a Ca^2+^-dependent manner in the damaged or diseased brain.

## Evidence Questioning Gliotransmission in Healthy Brain Tissue

### What is gliotransmission?

The process of gliotransmission has been defined as analogous to neurotransmission, except that the source of the transmitters is glia (i.e., astrocytes) rather than neurons (Volterra and Meldolesi, 2005). The other properties are essentially the same: a fast, Ca^2+^-dependent exocytosis of transmitters resulting in stimulation of synaptic receptors and acute synaptic modulation.

### Mechanisms of astrocyte Ca^2+^ elevations

It is first important to point out that there is no firm definition of an astrocyte. If the definition is exclusively morphological, i.e., highly ramified star-shaped cells, then the so-called “complex” glia could be included as an astrocyte subtype (see Nishiyama et al., 2005). This broad classification results in considerable heterogeneity in the astrocyte population and can be problematic when considering sources of Ca^2+^ elevations in astrocytes. Complex glia, so named because of their complex current pattern evoked using voltage-step protocols, have also been referred to as GluR cells due to their expression of functional ionotropic glutamate receptors (iGluRs; Matthias et al., 2003; Jabs et al., 2005). Evidence suggests that the AMPA iGluRs expressed by complex glia and Bergmann glia of the cerebellum are of the Ca^2+^-permeable variety, thereby providing a source for fast focal Ca^2+^ elevations in these cells (Lin and Bergles, 2004; Lin et al., 2005; Piet and Jahr, 2007; Hamilton et al., 2010). In developing brain tissue, complex glia also include immature passive astrocytes (Walz, 2000; Zhou et al., 2006), but in the mature brain they appear to be a unique cell population called NG2^+^ glia due to their surface expression of NG2 proteoglycan. NG2^+^ glial cells have a star-shaped morphology and do not fire action potentials, but otherwise differ from the subpopulation of mature passive astrocytes in nearly every other way (reviewed in Lin and Bergles, 2002). Importantly, there is little convincing evidence to date that passive astrocytes in intact tissue express neurotransmitter-gated ionotropic receptor types that could provide a source for fast local Ca^2+^ elevations. Because of these differences, inclusion of complex glia or specialized astrocytes such as Bergmann glia in the sampled population of “astrocytes” could lead to very different conclusions about astrocyte signaling properties. In this review, for clarity our definition of astrocyte includes the protoplasmic passive subtype only, which are the glial fibrillary acidic protein (GFAP)-expressing glial cells that express glutamate transporters, are coupled by gap junctions, and whose processes envelop synapses and form endfeet on the cerebrovasculature.

**Figure 1:**
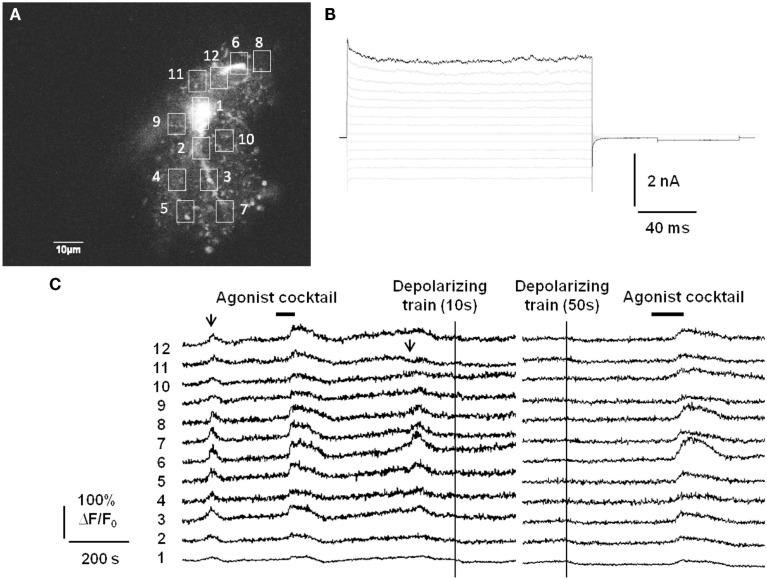
**Passive astrocytes do not express functional voltage-gated calcium channels**. **(A)** Astrocytes in stratum radiatum of acute hippocampal slices were patch-clamped with a small pipette tip (7–9 MΩ) in order to limit dialysis of the pipette contents during the recording. Included in the pipette was 150 μM of the cell impermeant Ca^2+^ indicator Oregon Green BAPTA-1 (OGB-1). **(B)** Passive mature astrocytes were characterized by the absence of voltage-gated currents evoked by a voltage-step protocol (−180 to +80 mV in 20 mV increments). **(C)** In 6/10 astrocytes from 10 slices, despite whole-cell voltage clamp the astrocytes exhibited spontaneous Ca^2+^ elevations (arrows) and evoked Ca^2+^ responses to an agonist cocktail consisting of 10 μM each of the G_q_ GPCR agonists histamine, carbachol, and ATP. However, strong depolarizing trains (−90 to 0 mV at 1 Hz for 10 or 50 s) through the patch pipette did not produce any increases in fluorescence, indicating a lack of voltage-gated Ca^2+^ channels in astrocytes. The remaining 4/10 astrocytes did not respond to depolarization or agonist cocktail with Ca^2+^ elevations, but did respond to agonist cocktail after pipette removal. This is presumably an effect of dialysis of the cell by the whole-cell recording pipette.

In addition to lacking Ca^2+^-permeable ligand-gated ion channels, there is also evidence that astrocytes do not express voltage-gated calcium channels (VGCCs; Carmignoto et al., 1998), although the expression of VGCCs in passive astrocytes may still be a matter of debate. We have assayed for functional expression of astrocytic VGCCs as well by applying strong trains of depolarizing stimuli via whole-cell patch pipette (Figure 1). The depolarizing stimuli did not evoke Ca^2+^ elevations anywhere in the astrocyte, which nevertheless exhibited spontaneous Ca^2+^ elevations and responded to bath application of a cocktail of agonists to endogenous G_q_ GPCRs. In summary, it does not appear that astrocytes possess a fast source of Ca^2+^ elevations capable of responding to neuronal inputs on a millisecond timescale. This also distinguishes gliotransmission from neurotransmission, which is triggered by the rapid gating of presynaptic VGCCs located adjacent to a readily releasable pool of synaptic vesicles.

The source of astrocyte Ca^2+^ elevations appears to be almost exclusively G_q_ GPCR activated, inositol 1,4,5-triphosphate receptor (IP_3_R)-sensitive intracellular stores, involving the canonical phospholipase C (PLC)/IP_3_ pathway. Upon G_q_ GPCR activation, PLC hydrolyzes the membrane lipid phosphatidylinositol 4,5-bisphosphate (PIP_2_) to generate diacylglycerol (DAG) and IP_3_, leading to IP_3_R activation and Ca^2+^ release from the endoplasmic reticulum (ER). The lines of evidence for G_q_ GPCR-mediated Ca^2+^ sources in astrocytes are multiple. First, there is strong evidence that astrocytes express G_q_ GPCRs for endogenous neurotransmitters (Porter and McCarthy, 1997) that can be activated by bath application of various agonists (e.g., Shelton and McCarthy, 2000). Second, Ca^2+^ elevations evoked by neuronal afferent stimulation *in situ* or by sensory stimulation *in vivo* are significantly inhibited by application of specific G_q_ GPCR antagonists (Porter and McCarthy, 1996; Perea and Araque, 2005; Wang et al., 2006). Third, spontaneous astrocytic Ca^2+^ transients are inhibited by the G-protein inhibitor GDP-βs, the IP_3_R antagonist heparin, or by depletion of internal Ca^2+^ stores using thapsigargin (Nett et al., 2002; Di Castro et al., 2011). Last, spontaneous and evoked astrocyte Ca^2+^ elevations are almost completely abolished by removal of the astrocyte-specific IP_3_R, the IP_3_R type 2 (IP_3_R2; Petravicz et al., 2008; Agulhon et al., 2010; Di Castro et al., 2011; Takata et al., 2011; Navarrete et al., 2012). Interestingly, one study has reported small residual and infrequent Ca^2+^ elevations remaining in astrocytic fine processes in IP_3_R2 knockout mice (Di Castro et al., 2011). The source of these Ca^2+^ elevations remains to be determined, but they may be G_q_ GPCR-independent and play a role in setting basal or resting Ca^2+^ levels in astrocytes (see below). Nevertheless, taken together the above evidence indicates that both evoked and spontaneous Ca^2+^ elevations in astrocytes are driven almost exclusively by G_q_ GPCR-coupled release from intracellular stores.

### Are astrocytic G_q_ GPCR-mediated Ca^2+^ elevations sufficient to induce gliotransmitter release?

Early work demonstrating gliotransmission in astrocyte-neuron co-cultures provided evidence that Ca^2+^ was “necessary and sufficient” for this process (Araque et al., 1998, 1999; Parpura and Haydon, 2000). Around this same period of time the emerging evidence that astrocytes *in situ* expressed numerous G_q_-coupled metabotropic receptor types that could be activated by neuronal activity set the stage for the concept of gliotransmission; i.e., that spillover of synaptically released neurotransmitter stimulates astrocytic G_q_ GPCR signaling cascades, resulting in astrocytic Ca^2+^ elevations that reciprocally and acutely modulate synaptic transmission through release of gliotransmitters (Volterra and Meldolesi, 2005; Halassa et al., 2007). More recent work has called into question the Ca^2+^-dependency of gliotransmission. To test the hypothesis that physiologically relevant astrocyte Ca^2+^ elevations results in gliotransmission required that astrocytic G_q_ GPCRs actually be stimulated. This was problematic in intact tissue since bath application of G_q_ GPCR agonists such as the group I mGluR agonist DHPG directly stimulates metabotropic glutamate receptors (mGluRs) on both astrocytes and neurons as well as other cell types including microglia (Pocock and Kettenmann, 2007; Farso et al., 2009). Therefore, transgenic tools were developed to selectively stimulate or eliminate astrocytic G_q_ GPCR-mediated Ca^2+^ elevations (Fiacco et al., 2007; Petravicz et al., 2008; Agulhon et al., 2010). Surprisingly, selective stimulation or removal of astrocyte G_q_ GPCR-mediated Ca^2+^ elevations had no effect on CA1 pyramidal neuron excitatory synaptic transmission and hippocampal short- and long-term plasticity (LTP). These findings suggested that astrocyte Ca^2+^ elevations are not sufficient for gliotransmission in acute hippocampal slices. The results of these studies have already been well-documented as they have led to considerable debate as to whether the mechanisms of gliotransmission, as they have been conceived, are physiologically relevant (Kimelberg, 2007; Hamilton and Attwell, 2010; Kirchhoff, 2010; Smith, 2010).

What could be the source of activity-induced astrocyte Ca^2+^ elevations responsible for gliotransmission then, if it is not from iGluRs, VGCCs, or G_q_ GPCRs? It has been recently pointed out that, due to the tiny size of fine astrocyte processes (20–30 nm) surrounding synapses, the relevant Ca^2+^ elevations involved in gliotransmission may be too small, too fast, and too local to detect using available Ca^2+^ indicators and two-photon imaging methods (Rusakov et al., 2011). However, these putative Ca^2+^ sources would not likely include G_q_ GPCRs/IP_3_Rs, as the 20- to 30-nm, thin sheet-like processes of astrocytes frequently surrounding synapses are most often devoid of ER (Peters et al., 1991). Furthermore, numerous reports have provided evidence to suggest that only very large astrocyte Ca^2+^ elevations evoked using strong afferent stimulation of multiple inputs or uncaging of IP_3_ or Ca^2+^ may be sufficient to release sufficient gliotransmitter from diffusely scattered synaptic-like microvesicles to overcome reuptake by astrocytic and neuronal transporters to stimulate neuronal receptors (Fiacco and McCarthy, 2004; Takata et al., 2011; Navarrete et al., 2012). A more intriguing possibility is that gliotransmitter release is dependent on other signaling molecules produced by activated microglia and reactive astrocytes in addition to astrocyte Ca^2+^ (e.g., Santello et al., 2011), as will be discussed further below.

**Figure 2:**
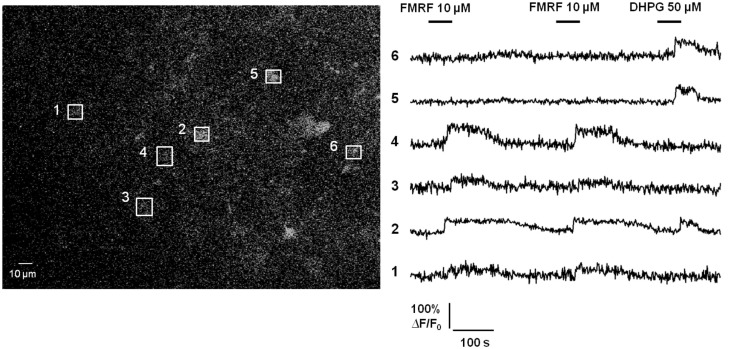
**Propagating intercellular Ca^2+^ waves are unlikely to occur between astrocytes in acute hippocampal slices from healthy brain**. Astrocytes in stratum radiatum of hippocampal slices from MrgA1 G_q_ GPCR transgenic mice were bulk-loaded with Ca^2+^ Green 1-AM indicator dye as described previously (Fiacco et al., 2007; left panel). Boxed regions of interest over individual astrocyte somata match numbered Ca^2+^ traces (right panel). Stimulation with either 10 μM FMRF or 50 μM DHPG evoked Ca^2+^ elevations in 56/75 (75%) or 67/75 (89%) of astrocytes, respectively, through activation of MrgA1Rs or group I mGluRs (*n* = 9 slices). In total, 50/75 (67%) of astrocytes responded to both agonists. However, 24/75 (32%) of astrocytes responded to one, but not the other, agonist. These results suggest that despite strong Ca^2+^ elevations in many astrocytes, the gliotransmitter ATP is not released in sufficient quantity to stimulate purinergic P2Y G_q_ GPCRs on adjacent astrocytes.

Additional evidence questioning Ca^2+^-dependent release of gliotransmitters by astrocytes in healthy brain tissue is the lack of propagating intercellular astrocyte Ca^2+^ waves. The original report of intercellular astrocyte Ca^2+^ waves in cultured astrocytes created quite a bit of excitement as it was postulated as a mechanism for long-distance signaling in the brain by an otherwise electrically non-excitable cell type (Cornell-Bell et al., 1990). Later work revealed that the mechanism underlying this phenomenon is release of the gliotransmitter ATP, which binds P2Y purinergic G_q_ GPCRs on adjacent astrocytes resulting in Ca^2+^ liberation from internal stores, ATP release, and thus wave regeneration and propagation (Cotrina et al., 2000; Arcuino et al., 2002; Bowser and Khakh, 2007). Propagating glial Ca^2+^ waves have also been observed in the white matter of neonatal brain tissue (Schipke et al., 2002) and in the retina (Newman, 2001), but not in cortical gray matter astrocytes *in situ* or *in vivo* (Fiacco and McCarthy, 2006; Kuchibhotla et al., 2009). We have performed additional experiments to assay for Ca^2+^ wave propagation between astrocytes using acute hippocampal slices from transgenic mice in which a large percentage of astrocytes selectively express the MrgA1 G_q_ GPCR (Fiacco et al., 2007; Figure 2). The design of these experiments is very simple: astrocytes were bulk-loaded with Ca^2+^ indicator and the MrgA1 receptor agonist FMRF and the group I mGluR agonist DHPG were applied in succession. Most astrocytes respond with characteristic long duration Ca^2+^ elevations to both agonists, but occasionally there are astrocytes that respond only to one agonist but not the other. Response to one agonist serves as a positive control that the astrocytes are viable. This is evidence against Ca^2+^-dependent release of ATP by astrocytes, because it is expected that astrocytes not expressing the MrgA1R or group I mGluRs would also have Ca^2+^ elevations due to the high amounts of ATP being released by the surrounding astrocytes. These data suggest that the only astrocytes capable of producing Ca^2+^ elevations are those cells that express the G_q_ GPCR being stimulated by the bath-applied agonist. It should be pointed out that cells expressing MrgA1R Ca^2+^ responses have been electrophysiologically confirmed as passive astrocytes in 100% of cases (*n* > 50 cells; unpublished observations). In summary, the propagating intercellular Ca^2+^ wave is clear evidence for astrocytic release of gliotransmitters, evidence which is lacking in astrocytes from healthy tissue *in situ* or *in vivo*. However, as will be discussed below, in the diseased or damaged brain propagating astrocytic Ca^2+^ waves are evident, suggesting that changes occurring in reactive astrocytes are permissive for gliotransmitter release.

### Basal (resting) Ca^2+^ vs. activity-evoked Ca^2+^ elevations

Many studies concluding a role for gliotransmission have used the Ca^2+^ chelators BAPTA and/or EGTA in an effort to block astrocytic G_q_ GPCR Ca^2+^ elevations. These manipulations have produced clear effects on neuronal activity, suggesting that activity-induced astrocyte Ca^2+^ elevations modulate neuronal activity by gliotransmission. It has been difficult to reconcile these findings with those using IP_3_R2 knockout mice, in which astrocytic G_q_ GPCR Ca^2+^ elevations are also abolished but without a corresponding effect on hippocampal excitatory synaptic activity or NMDA receptor-dependent LTP (for example, compare Agulhon et al., 2010 with Henneberger et al., 2010). Exciting new work has provided a potential explanation for this discrepancy (Shigetomi et al., 2012). Using a membrane-tethered genetically encoded Ca^2+^ indicator (Lck-GCaMP3), spotty Ca^2+^ signals were observed in cultured astrocytes that were not dependent on Ca^2+^ release from internal stores. Rather, these signals seemed to be dependent on extracellular influx of Ca^2+^ through transient receptor potential A1 (TRPA1) channels on the astrocyte membrane. These fast, focal Ca^2+^ elevations were shown to contribute to, and set resting Ca^2+^ levels in astrocytes. Could these channels represent the Ca^2+^ source required for gliotransmission? It cannot be ruled out; however, they seemed to open in an activity-independent manner and affect synaptic activity in hippocampal interneurons by a mechanism other than gliotransmission, i.e., by regulating the functional expression of astrocytic GABA transporters at the plasma membrane. It will be interesting in future work to determine if activity-independent Ca^2+^ influx setting basal Ca^2+^ levels is important for tonic release or uptake of gliotransmitters to set ambient concentrations of ATP/adenosine, d-serine, or glutamate (Jabaudon et al., 1999; Cavelier and Attwell, 2005; Cavelier et al., 2005; Le Meur et al., 2007). Together, these findings suggest that caution should be exercised in the interpretation of experiments dialyzing the astrocytic syncytium with Ca^2+^ chelators such as BAPTA and/or EGTA, which will not only block activity-induced G_q_ GPCR Ca^2+^ elevations but also clamp resting Ca^2+^ levels.

### Questionable timing for gliotransmission to acutely modulate synaptic transmission: Astrocytes as gliomodulators or “re-suppliers”

**Figure 3:**
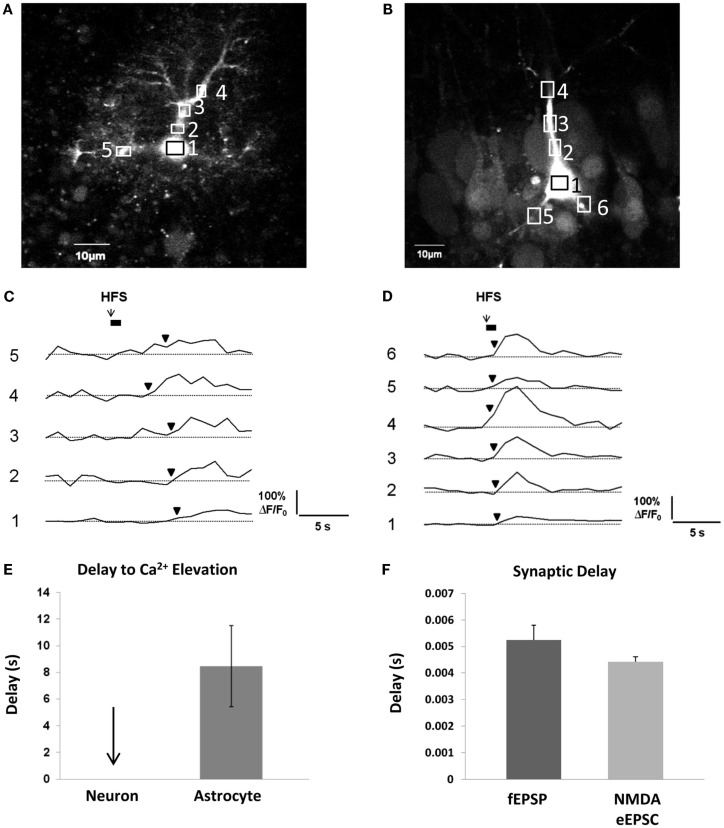
**Delay between Schaffer collateral stimulation and evoked astrocyte or neuronal Ca^2+^ responses and AMPA and NMDA receptor currents**. **(A,C)** Astrocyte in stratum radiatum **(A)** and CA1 pyramidal neuron **(B)** each filled with 150 μM OGB-1 Ca^2+^ indicator dye. Boxed regions of interest in subcellular compartments correspond to the fluorescence traces in **(C,D)**. Boxes were placed all over the visible astrocyte but only a few are shown for clarity. Cells were given 10 min to recover after removal of the whole-cell patch clamp pipette prior to Schaffer collateral stimulation. The stimulating electrode was placed 75 μm from the recorded cells. **(C,D)** Schaffer collateral stimulation at 50 Hz for 1s produced astrocytic Ca^2+^ elevations after ~3 s in the first responding process of this specific astrocyte **(C)**, while neuronal Ca^2+^ elevations occurred almost instantaneously in proximal neuronal compartments in this example as well as all other recorded neurons (*n* = 4) **(D)**. **(E)** Astrocyte Ca^2+^ elevations occurred on average after ~8 s in the first responding compartment (*n* = 4 cells). Response initiation times were defined as the first data point that preceded two successive data points that were ≥3 S.D. above the mean baseline noise. We have observed that the astrocytic Ca^2+^ responses to the first stimulation are faster and more reliable than subsequent stimulations, so only the responses to the first stimulation were calculated. **(F)** In the same conditions, fEPSPs (*n* = 5 slices) and whole-cell CA1 neuronal NMDA eEPSCs (*n* = 16 cells, 16 slices) were evoked after ~5 and 4 ms, respectively, in response to single depolarizing pulses (0.05 Hz) to the Schaffer collaterals.

In order for gliotransmission to reciprocally affect synaptic events occurring on a millisecond timescale, it needs to occur along a similar timescale. As discussed above, evidence to date indicates that metabotropic G_q_ GPCRs are the prevailing mechanism behind activity-induced astrocytic Ca^2+^ elevations. Because the pathway to Ca^2+^ elevations involves a metabotropic signaling cascade, there is a considerable delay between neurotransmitter binding the receptor and the ensuing Ca^2+^ response. In our own recordings at room temperature in acute hippocampal slices, the time interval – on average – between a 50-Hz train of Schaffer collateral stimulation and the first responsive astrocytic compartment (almost always a small to medium-sized process) is 8 s (Figures 3A,C,E). This of course, is an average, and there may be some small compartments and/or a small subset (5%) of astrocytes capable of faster responses (Winship et al., 2007; Panatier et al., 2011). The remaining astrocytic compartments (encompassing the vast majority of the cell volume) take longer to respond due to the slow intracellular wave propagation rate of astrocyte Ca^2+^ (5–15 μm/s; Yagodin et al., 1994; Fiacco and McCarthy, 2006; Fiacco et al., 2007). Some of the delay to the initial astrocyte Ca^2+^ response can be attributed to the kinetics of the Ca^2+^ indicator dye converting the binding of Ca^2+^ to a fluorescence signal and to the spatial and temporal resolution of optical detection, but this can be taken into account by recording neuronal Ca^2+^ signals using the same fluorescence indicator and microscope settings. In contrast to astrocytic Ca^2+^ elevations, neuronal Ca^2+^ elevations using the same settings appear to occur instantaneously (Figures 3B,D,E). In the same preparation and in the same conditions, the delay to evoke excitatory field potentials (fEPSPs) from a single depolarizing pulse to the Schaffer collaterals is ~5 ms, while whole-cell NMDA receptor-mediated eEPSCs are evoked after ~4 ms (Figure 3F). Calcium elevations in the dendritic spines due to NMDA receptor activation are detected a little over 10 ms after photolysis of extracellular caged glutamate (Bloodgood and Sabatini, 2007), while elevations in the proximal dendrites generated by the opening of VGCCs gated by back-propagating action potentials occur within 100–200 ms (Fedirko et al., 2007). Therefore, detection issues cannot account for slow Ca^2+^ response times of astrocytes. Recording at room temperature will also slow down astrocyte Ca^2+^ response times, since the activity rates of effector proteins and enzymes in the G_q_ GPCR signaling cascade will be reduced compared to physiological temperature. However, even *in vivo*, astrocyte cell bodies respond to sensory or motor stimulation after a delay of ~2–10 s, with Ca^2+^ increases in the processes occurring ~1 s earlier than those in the soma (Wang et al., 2006; Dombeck et al., 2007; Schummers et al., 2008; Navarrete et al., 2012). Overall, the bulk of the evidence indicates that activity-induced astrocytic G_q_ GPCR Ca^2+^ responses in processes occur on a timescale of seconds. This is a 1000-fold slower than synaptic responses.

Now, as an example, we will consider this timing in the context of release of the gliotransmitter d-serine by astrocytes. d-serine is a required co-agonist of the NMDA receptor – without d-serine bound the NMDA receptor ion channel cannot open (Johnson and Ascher, 1987; Mothet et al., 2000). The complete cycle of d-serine synthesis, release, uptake, and enzymatic breakdown is not yet fully understood, but most evidence suggests that there are high – although not completely saturating – concentrations of extracellular d-serine in most forebrain areas (Miller, 2004; Verrall et al., 2010). This leaves room for activity-dependent modulation of NMDA receptor function by regulated release of d-serine. For the sake of this discussion we must first assume that astrocytes are capable of producing and secreting d-serine, which has recently come under question. d-serine was originally thought to be produced and secreted only by astrocytes due to astrocyte-specific expression of the synthesizing enzyme serine racemase (SR; Wolosker et al., 1999). More recent data using different antibodies to SR and selective knockout studies suggest that SR is a neuronal enzyme (Kartvelishvily et al., 2006; Miya et al., 2008; Benneyworth et al., 2012). The recent study by Benneyworth et al. (2012) does suggest that a small portion of extracellular d-serine might be supplied by astrocytes.

**Figure 4:**
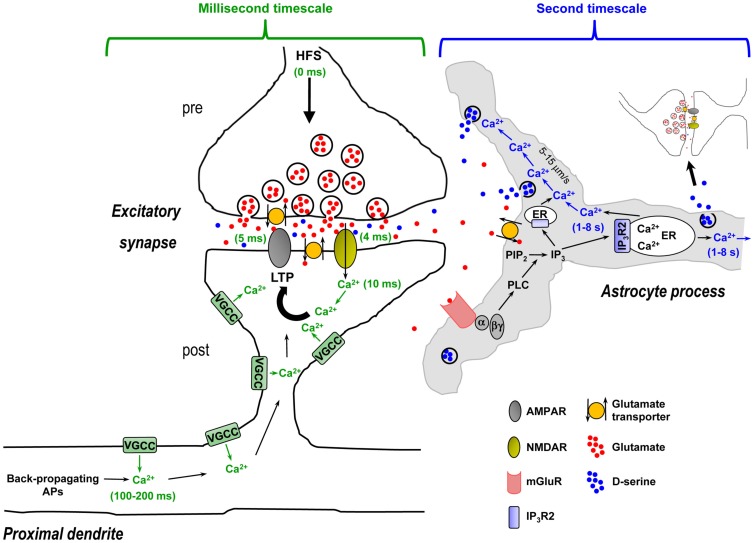
**Time course of synaptic mechanisms leading to the induction of NMDA receptor-mediated LTP vs. time course of activity-evoked G_q_ GPCR Ca^2+^-mediated d-serine release from astrocytes in CA1 hippocampus: millisecond timescale mechanisms vs. second timescale mechanisms**. Left panel, Induction of LTP requires: (i) High-frequency presynaptic stimulation (HFS) to release the neurotransmitter glutamate – beginning of HFS is noted as 0 ms time point; (ii) Depolarization of the postsynaptic compartment by glutamate-mediated AMPA receptor activation allowing Mg^2+^ block relief of the NMDA receptors, and activation of NMDA receptors by glutamate and necessary co-agonist d-serine (4–5 ms time points); (iii) Ca^2+^ entry through NMDA receptor channel (10 ms time point). This resulting rise in Ca^2+^ is crucially important to the induction of LTP and may be amplified by concurrent activation of VGCCs. At 100–200 ms time point, Ca^2+^ is elevating in the proximal apical dendrite due to back-propagating APs that might act as a retrograde signal to modulate LTP to some extent. All of these steps happen over a timescale of milliseconds and are represented in green. Right panel, HFS-evoked release of d-serine from astrocytes requires: (i) Spillover of presynaptically released glutamate from the synapse, reaching sufficient levels to overcome glutamate clearance by neuronal and astrocytic glutamate transporters; (ii) Glutamate binding and activation of astrocytic mGluRs on astrocyte processes enveloping synapses, which leads to PLC-mediated formation of IP_3_; (iii) Ca^2+^ release from ER upon IP_3_R2 receptor channel activation by IP_3_ binding (1–8 s time point – represented in blue). Note that in the astrocyte sheet-like processes surrounding synapses that are frequently 20–30 nm thick, the presence of ER might be limited and the IP_3_ must diffuse into thicker astrocyte processes where ER components are more likely present; (iiii) Intercellular Ca^2+^ wave propagation (5–15 μm/s) that triggers exocytosis of the sparse d-serine containing synaptic-like vesicles encountered along the way. In this context, activity-evoked d-serine release from astrocytes occurs in a second timescale after the beginning of HFS and therefore cannot be the source of the required co-agonist necessary for NMDA receptor activation, which occurs within 4–5 ms. Activity-induced and G_q_ GPCR Ca^2+^-mediated release of d-serine from astrocytes seems to be more optimally suited to modulate subsequent neuronal activity (i.e., involving gliomodulation as opposed to acute “gliotransmission”) or plays a role to “re-supply” ambient levels of extracellular d-serine, which is partially depleted through HFS. Abbreviations: AP, action potential; α, G_q_ α subunit; βγ, G_q_ βγ subunits; ER, endoplasmic reticulum; IP_3_, inositol 1,4,5-triphosphate; IP_3_R2, astrocyte-specific IP_3_ receptor type 2; LTP, long-term potentiation; PIP_2_, inositol bisphosphate; PLC, phospholipase C; VGCC, voltage-gated Ca^2+^ channel.

Activity-dependent release of d-serine by astrocytes has been reported to be required for hippocampal LTP as well as LTP of local field potentials in the somatosensory cortex (Henneberger et al., 2010; Takata et al., 2011). The idea is that spillover of glutamate or glutamate + acetylcholine (ACh) from repetitive stimulation of neuronal afferents activates astrocytic mGluRs or mGluRs + metabotropic ACh receptors (mAChRs) to trigger Ca^2+^-dependent d-serine release from astrocytes, which is then necessary for postsynaptic NMDA receptor activation and LTP. However, if we walk through the steps in this process it becomes evident that the timing is problematic (Figure 4 – for simplicity only hippocampal NMDA receptor-mediated LTP is depicted). First, presynaptically released glutamate during the high-frequency stimulus (HFS) train crosses the narrow (20 nm) synaptic cleft and binds postsynaptic NMDA and AMPA receptors. In approximately 4–5 ms these channels open and pass postsynaptic current, and postsynaptic Ca^2+^ increases *via* entry through the NMDA receptor channel are observed after 10 ms (Bloodgood and Sabatini, 2007). Within 100–200 ms, Ca^2+^ is already elevating in the proximal apical dendrites due to back-propagating action potentials (Fedirko et al., 2007). During the HFS train the concentration of glutamate/ACh builds up and spills out of the synapse, reaching sufficient levels to overcome glutamate clearance or ACh breakdown to bind and activate astrocytic mGluRs or mAChRs. The activated G_q_ GPCRs exchange GDP for GTP and the α and βγ subunits dissociate. The α subunit then activates the enzyme phospholipase C (PLC) in the astrocyte membrane. Next, PLC mediates the conversion of inositol bisphosphate (PIP_2_) to IP_3_ and diacylglycerol (DAG). IP_3_ builds up in the astrocytic cytosol and binds its target receptor, IP_3_R2, on the ER. Activation of the IP_3_R2 results in channel opening and Ca^2+^ efflux from the ER into the cytosol, detected by the fluorescence indicator a few seconds after neuronal afferent stimulation. The locally elevated Ca^2+^ propagates into adjacent astrocytic compartments including the 20- to 30-nm sheet-like processes frequently surrounding synapses at a rate of 5–15 μm/s, where it encounters along the way small and diffuse synaptic-like microvesicles which are triggered to exocytose and release d-serine into the extrasynaptic space. The extracellular d-serine presumably reaches a sufficient concentration to percolate back into the synapse to bind synaptic NMDA receptors, permitting NMDA receptor channels to open to pass current and elevate postsynaptic Ca^2+^.

The obvious problem with this scenario is that the requirement for astrocytic d-serine as a necessary co-agonist at the NMDA receptor occurred a 1000 times earlier in relative synaptic time (Figure 4). This scenario does not take into account the rise time of astrocyte Ca^2+^ elevations, which is often seconds long (Wang et al., 2006; Henneberger et al., 2010; Navarrete et al., 2012). The very observation that NMDA receptor currents are evoked in ~4 ms and postsynaptic Ca^2+^ elevates shortly thereafter indicates that the d-serine required for NMDA channel opening was already available, as suggested by the relatively high ambient d-serine levels in the synapse (Mothet et al., 2000). The remaining activity-dependent release of d-serine may come from the presynaptic or postsynaptic neuronal compartments, as reported in a recent study (Rosenberg et al., 2010). The advantage provided by this mechanism is that ambient and activity-induced release of d-serine will be available as a required co-agonist at the same time that presynaptically released glutamate is binding the NMDA receptor. Astrocytes, known to maintain ionic and neurotransmitter homeostasis (Kimelberg, 2007), might instead participate in setting ambient d-serine levels in a manner dependent on resting Ca^2+^. In this context, it seems conceivable that reducing or clamping resting Ca^2+^ levels in astrocytes using intracellular Ca^2+^ chelators minutes before HFS could decrease overall d-serine levels and LTP. Such a hypothesis, if validated, could reconcile discrepancies between studies (Agulhon et al., 2010; Henneberger et al., 2010).

Because of the slow timing of metabotropic receptor-evoked astrocyte Ca^2+^ elevations, astrocytes seem to be more optimally suited to modulate subsequent neuronal activity or play a role as “re-suppliers” as opposed to acute modulators. Is this gliotransmission? Arguably not, based on the current definition of gliotransmission. The term “gliomodulation” may be more appropriate and better distinguish this process from neurotransmission. Interestingly, Rosenberg et al. (2010) found that extracellular d-serine is reduced minutes after its initial increase following neuronal depolarization. Astrocyte Ca^2+^ may then participate in re-setting ambient levels of d-serine following depletion through repetitive neuronal activity. Astrocytes as re-suppliers can also be considered in the case of cerebrovascular coupling. As this process is also thought to be regulated by activity-induced astrocyte G_q_ GPCR Ca^2+^ (Simard et al., 2003; Zonta et al., 2003; Mulligan and MacVicar, 2004), astrocytes are well-positioned to re-supply neurons with metabolites and oxygen after these substrates are depleted during repetitive neuronal activity.

## Evidence Supporting Ca^2+^-Dependent Gliotransmitter Release in the Early Steps of the Inflammatory Process and in the Diseased or Damaged Brain

### Reactive astrocytes

In the previous section we discussed evidence questioning the existence of gliotransmission in healthy brain tissue. In this section we will review data suggesting that activated microglia and reactive astrocytes engage in a partnership for Ca^2+^-dependent gliotransmitter release. First, what is a reactive astrocyte? Reactive astrocytes or reactive astrogliosis can be defined as a graded progression of molecular, cellular, and functional changes that range from subtle alterations in gene expression to glial scar formation (Hamby and Sofroniew, 2010). Reactive astrocytes are a hallmark of nearly all brain pathologies, including traumatic brain injury, stroke, ischemia, infectious disease, neuroinflammatory and neurodegenerative disease, epilepsy, brain tumors, and possibly even more subtle disorders such as schizophrenia, migraine, and depression. The list of changes that have been documented to occur in reactive astrocytes is extensive, and includes hypertrophy (Sofroniew, 2009), upregulated expression of GFAP (Pekny and Nilsson, 2005), altered expression of: mGluRs (Aronica et al., 2003), glutamate transporters and glutamine synthetase (Pardo et al., 2006; Ortinski et al., 2010), ephrins and their receptors (Goldshmit et al., 2006), aquaporin-4 (AQP4) and potassium channels (Binder and Steinhauser, 2006), and production and secretion of inflammatory molecules (e.g., TNFα, SDF-1α, PGE_2_) and oxygen free radicals (Lucas et al., 2006; Peng et al., 2006; Farina et al., 2007; Brambilla et al., 2009; Steele and Robinson, 2012).

One of the persistent problems in understanding astrocytic involvement in progression of changes occurring in the diseased or damaged brain is establishing cause-and-effect relationships. It seems that research is beginning to move past cataloging changes that are present in damaged tissue to carefully examining the initial sequence of events occurring in the progression of disease before overt neuroanatomical changes or onset of behavioral symptoms (Colangelo et al., 2012). Accumulating evidence is suggesting that microglia, the brain’s resident immune cells, are activated first, and through release of ATP and inflammatory mediators including interleukin-1 (IL-1), TNFα, or PGE_2_, subsequently trigger astrocytic activation (Bezzi et al., 2001; Ikeda-Matsuo et al., 2005; Liu et al., 2011; Machado et al., 2011; Colangelo et al., 2012; Pascual et al., 2012). Activated (reactive) astrocytes then increase their production and secretion of SDF-1α (Bajetto et al., 1999; Peng et al., 2006), TNFα, and/or PGE_2_ (Bezzi et al., 2001; Alvarez et al., 2010) which, in conjunction with Ca^2+^, promotes gliotransmitter (ATP, glutamate) release. All of these molecules may act in concert through complex autocrine/paracrine or astrocyte-microglial signaling pathways to generate and amplify propagating intercellular astrocyte Ca^2+^ waves and increase neuronal excitability (Bajetto et al., 1999; Cotrina et al., 2000; Bezzi et al., 2001; Han et al., 2001; Arcuino et al., 2002; Domercq et al., 2006; Bowser and Khakh, 2007; Santello et al., 2011; Pascual et al., 2012). These events are initiated rapidly, within seconds to minutes, suggesting that they may be among the early events underlying excitotoxic damage in disorders and diseases of the central nervous system.

### Propagating intercellular Ca^2+^ waves

As discussed above, evidence for intercellularly propagating Ca^2+^ waves among cortical gray matter astrocytes in healthy brain tissue *in situ* or *in vivo* is lacking (Fiacco and McCarthy, 2006; Kuchibhotla et al., 2009). On the contrary, dramatic changes in astrocytic Ca^2+^ homeostasis have been observed under pathological conditions (e.g., epilepsy or Alzheimer’s disease) *in situ* and *in vivo*, affecting the frequency, duration, and amplitude of astrocytic Ca^2+^ transients (Aguado et al., 2002; Hirase et al., 2004; Kuchibhotla et al., 2009). Powerful astrocytic intercellular Ca^2+^ waves were found to travel across the cortex in a mouse model of Alzheimer’s disease *in vivo* but not in wild type littermates (Kuchibhotla et al., 2009), suggesting that Ca^2+^ waves signal the existence of a pathological insult, as previously postulated (Fiacco and McCarthy, 2006; Scemes and Giaume, 2006). Long-distance and tetrodotoxin-insensitive astrocytic intercellular Ca^2+^ waves have also been described in spreading depression (Basarsky et al., 1998; Kunkler and Kraig, 1998; Peters et al., 2003; Chuquet et al., 2007), which is believed to occur in several neurological disorders including migraine (Hadjikhani et al., 2001; James et al., 2001), trauma (Fabricius et al., 2006), and stroke (Strong et al., 2002; Dreier et al., 2006; Fabricius et al., 2006), contributing to the death of compromised tissue (Busch et al., 1996; Nedergaard, 1996). In summary, in the diseased or damaged brain propagating astrocytic Ca^2+^ waves are evident, suggesting that changes occurring in reactive astrocytes may be permissive for Ca^2+^-dependent release of gliotransmitters (ATP).

### Requirement of activated microglia, reactive astrocytes and inflammatory molecules, and potential for therapeutic intervention

An intriguing possibility conferring pathological relevance to astrocytic release of gliotransmitters is that this process is actually dependent on other signaling molecules produced by activated microglia and reactive astrocytes in addition to Ca^2+^. A new report supports this hypothesis by showing that activated microglia recruit astrocytes to modulate neuronal activity early in the inflammatory process (Pascual et al., 2012). Activation of microglia using a proinflammatory toll-like receptor 4 ligand (TLR4), lipopolysaccharide (LPS), induced a rapid (within minutes) and transient (~10 min-long) increase in the frequency of excitatory synaptic events in acute hippocampal slices. The mechanism for this effect involved the release of ATP by microglia to activate metabotropic P2Y1 receptors (P2Y1Rs) on astrocytes, triggering glutamate release from astrocytes to modulate synaptic mGluRs. Because activation of microglia and alteration of neurotransmission are two early symptoms of most brain diseases, these findings support the idea that activated microglia are an upstream partner of astrocytes that transforms astrocytes into glutamate releasing cells, which may contribute to the initiation of bursting neuronal activity in the epileptic brain (Pascual et al., 2012) or other neurological disorders. Remarkably, the inflammatory mediators TNFα and PGE_2_ can also be co-released downstream of astrocytic ATP-induced P2Y1R activation, dramatically enhancing glutamate release from astrocytes, with TNFα action being a requirement for this glutamate response to occur (Domercq et al., 2006; Santello et al., 2011); although the requirement of TNFα is debated (Pascual et al., 2012). Even more striking is the observation that inflammatory molecules themselves can elicit a rapid (seconds) astrocytic release of glutamate without involvement of any G_q_ GPCR stimulation. SDF-1α, TNFα, or PGE_2_ by themselves are sufficient to induce glutamate release from astrocytes through selective activation of their respective receptors: G_i/o_ GPCRs (CXCR4), trimeric TNF receptors (TNFR), or G_i_/G_s_ GPCRs [prostaglandin E (EP) receptors; Bezzi et al., 1998, 2001; Sanzgiri et al., 1999; Cali et al., 2008]. Although understanding of the pathways downstream of CXCR4, TNFR, or EPs remains largely incomplete in astrocytes, it has been assumed that selective activation of one of these receptor types is sufficient to elicit a Ca^2+^-dependent exocytosis of glutamate from astrocytes. This assumption is based mainly on a study suggesting that CXCR4, TNFR, and EPs participate in the same sequence of events that ultimately leads to Ca^2+^-dependent exocytosis of glutamate, a response otherwise blocked by intracellular Ca^2+^ chelators or inhibitors of exocytosis (Bezzi et al., 2001). CXCR4 activation induces a complex ensuing signaling cascade involving extracellular release of TNFα from astrocytes, autocrine/paracrine TNFα-dependent signaling, PGE_2_ formation and secretion from astrocytes, autocrine/paracrine PGE_2_-dependent signaling, and finally glutamate release from astrocytes (Bezzi et al., 2001). In this context, Ca^2+^ is mobilized from internal stores following activation of CXCR4 (Zheng et al., 1999; Bezzi et al., 2001; Cali et al., 2008) and EP receptors (Sanzgiri et al., 1999), including G_i_ GPCR EP3 (Takemiya et al., 2011) and G_s_ GPCR EP2 receptors (Di Cesare et al., 2006; Hsiao et al., 2007). CXCR4- or EP-evoked glutamate release from astrocytes induces presynaptic NMDA receptor-dependent synaptic potentiation (Santello et al., 2011) or postsynaptic/extrasynaptic NMDA receptor-dependant slow inward currents (Sanzgiri et al., 1999). Interestingly, EP-mediated Ca^2+^ elevations spread through neighboring astrocytes as intercellular Ca^2+^ waves (Sanzgiri et al., 1999), strongly suggesting that not only glutamate, but also ATP is released (Cotrina et al., 2000; Arcuino et al., 2002; Bowser and Khakh, 2007) following EP activation. Notably, SDF-1α-induced CXCR4-mediated glutamate release is dramatically enhanced in the presence of reactive microglia, leading to pro-apoptotic neurotoxicity, a mechanism potentially involved in AIDS dementia complex (Bezzi et al., 2001; Rossi and Volterra, 2009). Not only astrocytes, but also microglia and neurons express CXCR4 (Tanabe et al., 1997; Asensio and Campbell, 1999; Kaul and Lipton, 1999), and activation of this receptor in microglia causes significant TNFα release. Microglial TNFα would then add to astrocytically released TNFα to amplify the release of glutamate from astrocytes.

**Figure 5:**
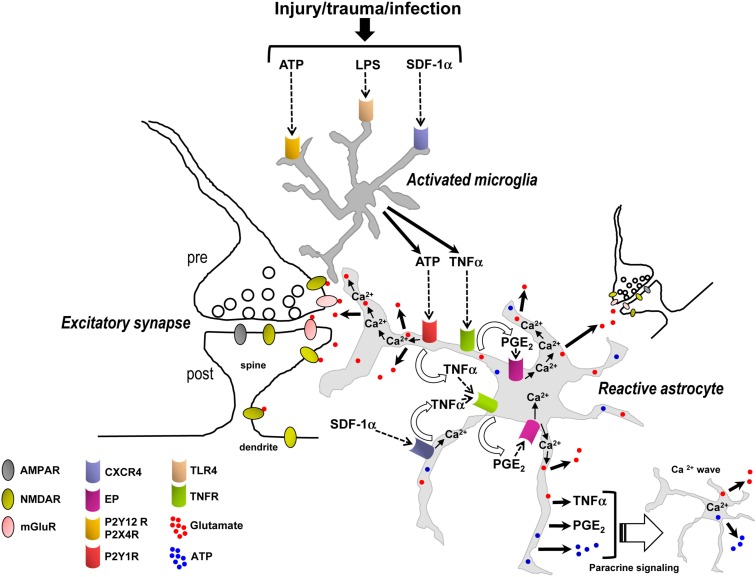
**Synergistic signaling transduction pathways involving activated microglia and reactive astrocytes and their inflammatory mediators that, in addition to astrocytic Ca^2+^, may induce the release of gliotransmitters (glutamate, ATP) in the early steps of the inflammatory process and in the diseased or damaged brain**. ATP, LPS, and/or SDF-1α are produced following acute and chronic pathological insults to the central nervous system, e.g., hypoxia, ischemia, epilepsy-associated seizures, mechanical injury and trauma, or infection (Bajetto et al., 2001; Bodin and Burnstock, 2001; Cook and McCleskey, 2002; Rivest, 2003; Qin et al., 2005). These mediators activate microglial purinergic receptors (P2Y12R, P2X4R; Haynes et al., 2006; Ohsawa and Kohsaka, 2011), TLR4 or CXCR4 as well as astrocytic CXCR4. Activated microglia then release ATP and/or TNFα to recruit astrocytes. In astrocytes, binding of ATP and SDF-1α – alone or in concert – to their G_q_ and G_i/o_ protein-coupled receptors (P2Y1R and CXCR4) stimulates Ca^2+^ mobilization from internal stores. P2Y1R and/or CXCR4-mediated intracellular Ca^2+^ elevations trigger an ensuing cascade of pathways involving the production and secretion of TNFα and PGE_2_, TNFR and EP activation, EP-induced Ca^2+^ elevations, and ultimately followed by the release of glutamate from astrocytes. For simplicity TNFα and PGE_2_ have been depicted as acting on the same astrocyte from which they are released; i.e., in an autocrine manner, although they can also act on neighboring astrocytes to propagate signals in a paracrine way and induce glutamate release. While astrocytic P2Y1R activation may also induce Ca^2+^-dependent release of glutamate without implication of TNFα, TNFα (from microglial and astrocytic origin), and PGE_2_ dramatically enhance the astrocyte glutamate response. The locally elevated Ca^2+^ due to activation of P2Y1R, CXCR4, and EPs, working alone or in concert, propagates some distance into astrocytic compartments including the sheet-like processes surrounding synapses where it encounters small and diffuse synaptic-like microvesicles which are triggered to exocytose glutamate into the extracellular space. Additionally, EP-mediated Ca^2+^ increases in astrocytes can also induce the release of ATP, which through autocrine/paracrine activation of astrocytic P2YRs evokes regenerative intercellular Ca^2+^ waves. Definitive proof for Ca^2+^-dependent astrocyte glutamate release to occur *via* exocytosis awaits further evidence. The overall glutamate release from astrocytes can occur within seconds to minutes of a triggering stimulus to increase ongoing pre- and postsynaptic mGluR- and/or NMDA receptor-mediated synaptic transmission, which may be a critical early step contributing to the initiation of neuronal signaling cascades leading to hyperexcitability, excitotoxicity, and cell death. Therefore the transduction pathways depicted in this figure could be potential targets for therapy of multiple neurological, neuroinflammatory, and neurodegenerative disorders.

The synergistic initiation of signaling transduction pathways involving microglia and astrocytes suggests that both cell types and their signaling pathways may be required, in addition to astrocytic Ca^2+^, to trigger the release of glutamate from astrocytes (Figure 5). The observation that activated microglia or inflammatory mediators cannot only induce, but also substantially amplify glutamate release from astrocytes confers pathological relevance to this process. Furthermore, the rapid (seconds to minutes) induction of these different pathways suggests that they may be critical early events contributing to initiation of neuronal signaling cascades leading to hyperexcitability, excitotoxicity, and cell death. Therefore these transduction pathways could be potential targets for therapy of multiple neurological, neuroinflammatory, and neurodegenerative disorders, including stroke, trauma, epilepsy, Alzheimer’s disease, Huntington’s disease, AIDS dementia complex, and amyotrophic lateral sclerosis. These and a growing list of other neurological disorders are now understood to share the final common destructive metabolic pathway of excitotoxicity, which is an excessive activation of neuronal glutamatergic receptors and associated signaling molecules (Shaw, 1993; Lau and Tymianski, 2010). Additionally, activated/reactive microglia and astrocytes, and production of inflammatory mediators is also another common element in most, if not all, neurological, neuroinflammatory, and neurodegenerative disorders (Lucas et al., 2006). Glial inflammatory transduction pathways leading to astrocytic Ca^2+^-dependent glutamate release could contribute to excitotoxicity, providing a new framework for elucidating the mechanistic basis of excitotoxicity and potentially improving upon existing medications.

Interestingly, in the inflammatory process involving activated microglia and reactive astrocytes, glutamate release from astrocytes may be mediated by Ca^2+^ elevations downstream of not only G_q_ (P2Y1R) GPCRs, but also G_i/o_ (CXCR4) or G_i_/G_s_ (EP) GPCRs. Additionally, the ligands of these receptors (ATP, SDF-1α, PGE_2_) and the ligand for TNFR (TNFα) appear to be mainly of microglial and astrocytic origin (Asensio and Campbell, 1999; Bajetto et al., 1999; Sanzgiri et al., 1999; Bezzi et al., 2001; Rostasy et al., 2003; Domercq et al., 2006; Peng et al., 2006; Rossi and Volterra, 2009; Santello et al., 2011; Pascual et al., 2012) and not of presynaptic origin; although, a contribution of ATP co-released with glutamate from neuronal afferents (Khakh, 2001) or of neuronal SDF-1α (Rostasy et al., 2003) cannot be excluded. The seconds to minutes timescale for the astrocytic glutamate response to occur fits with the timing expected of a metabotropic signaling pathway and also indicates that microglia can become activated, and astrocytes reactive, very quickly in response to a triggering stimulus (Figure 5). The data suggest that inflammation – in response to infection or injury, rather than synaptically induced stimulation of astrocytic GPCRs – is essential for triggering glutamate release from astrocytes to subsequently increase ongoing excitatory synaptic transmission (Figure 5). These emerging findings also need to be taken into consideration in the interpretation of studies that have used tetanus toxin to block glutamate exocytosis from astrocytes (Bezzi et al., 2001). Transport of cell surface connexin hemichannels or volume-activated Cl^−^ channels to the membrane via exosome trafficking may also be impaired by incubation in tetanus toxin (Proux-Gillardeaux et al., 2005), which could alter efflux of glutamate into the extracellular space by these alternate pathways (Malarkey and Parpura, 2008). Tetanus toxin would also presumably affect: (i) the transport of multidrug resistance protein 4 (MRP4) – a PGE_2_ export pump – to the membrane (Reid et al., 2003; Proux-Gillardeaux et al., 2005; Ronaldson et al., 2008) which could alter PGE_2_ release; or (ii) exocytosis of cytokine vesicular carriers which could decrease the secretion of inflammatory mediators (e.g., TNFα or other unidentified factors; Stow et al., 2009; Duitman et al., 2011). Thus, caution needs to be exercised in the interpretation of experiments using blockers of exocytosis or Ca^2+^ chelators in astrocytes (see discussion above) in combination with neuronal electrophysiological readouts, which may lead to misinterpretation of how astrocytes actually control extracellular glutamate levels in a Ca^2+^-dependent manner.

Glial inflammatory mediators such as SDF-1α or TNFα are expressed in the healthy brain, albeit at much lower levels than during inflammatory reactions and are thought to be implicated in regulating homeostatic brain functions (Vitkovic et al., 2000; Bajetto et al., 2001; Beattie et al., 2002; Stellwagen et al., 2005; Stellwagen and Malenka, 2006; Kaneko et al., 2008; Boulanger, 2009). Furthermore, Santello et al. (2011) reported that knockout of TNFα prevented P2Y1R Ca^2+^-dependent glutamate release from astrocytes in acute hippocampal slices from otherwise healthy mice. Therefore, constitutive levels of inflammatory mediators could also regulate to some extent astrocytic glutamate release in brain tissue from healthy mice. However, it is worth noting that astrocytic inflammatory mediator-dependent glutamate release has been obtained in cultured astrocytes, mixed microglia/astrocyte co-cultures or acute hippocampal slices (Bezzi et al., 1998, 2001; Sanzgiri et al., 1999; Domercq et al., 2006; Cali et al., 2008; Santello et al., 2011; Pascual et al., 2012). Microglia and astrocytes, by virtue of culturing conditions become activated (Cahoy et al., 2008; Zamanian et al., 2012), and their activity would therefore resemble more closely that which occurs in the late stages of inflammation. Acute slices, while offering the advantage of maintaining relationships between cell types and limiting reactive changes induced by culturing, are nevertheless also compromised to varying degrees. The slicing process invariably causes cellular and tissue damage including axotomy of afferent fibers (Coltman and Ide, 1996), accompanied by immediate release of ATP *via* cytolysis due to cell membrane damage, which may only be partially recovered during the subsequent incubation process (Bodin and Burnstock, 2001; Cook and McCleskey, 2002; Fiala et al., 2003). Central nervous system tissue can exhibit elevated ATP release for several hours after trauma (Wang et al., 2004). This form of lesion/trauma-induced injury is likely to cause activation of microglia (Nimmerjahn et al., 2005; Haynes et al., 2006; Ohsawa and Kohsaka, 2011) and astrocytes (Fiala et al., 2003; Neary et al., 2003) within the first minute of slicing. It is therefore reasonable to suggest that microglia and astrocytes in acute slices may be releasing higher levels of inflammatory mediators than they would do otherwise *in vivo*. This may make astrocytes more sensitive to stimulation by neurotransmitters or bath application of ATP, SDF-1α, TNFα, or PGE_2_. Astrocytic Ca^2+^-dependent glutamate release in these conditions may therefore most likely reflect responses of activated microglia and reactive astrocytes at the early stages of inflammation rather than physiological roles of presumed resting glial cells. In any case, differences in levels of TNFα and other inflammatory mediators between slice preparations could explain the variability between groups to produce or observe Ca^2+^-dependent glutamate release by astrocytes. Carefully controlled future studies *in vivo* may partially circumvent pathology associated with cell culture and acutely isolated brain slices, and help resolve whether astrocytic GPCR Ca^2+^-dependent glutamate release is relevant in physiological and/or pathological situations.

## Summary and Perspectives

A “calcium-centric” view has pervaded astrocyte research ever since the discovery of propagating astrocyte intercellular Ca^2+^ waves *in vitro* in the early 1990s (e.g., Cornell-Bell et al., 1990). While astrocyte Ca^2+^ increases undoubtedly play a role in certain aspects of astrocyte function, accumulating evidence suggests that there is more to astrocyte physiology than Ca^2+^ elevations. One of the most documented areas of astrocyte research over the past 20 years has been G_q_ GPCR Ca^2+^-dependent gliotransmitter exocytosis to acutely modulate the excitability of adjacent astrocytes and neurons. However, gliotransmission in healthy tissue has been called into question based on numerous findings. First, some studies have reported that selective stimulation or removal of G_q_ GPCR-dependent astrocyte Ca^2+^ elevations does not result in changes in neuronal synaptic transmission and plasticity, suggesting that astrocyte Ca^2+^ elevations are not sufficient to trigger the release of gliotransmitters (ATP, d-serine, glutamate). Second, new research suggests that basal or resting astrocyte Ca^2+^ levels need to be carefully considered when Ca^2+^ chelators are infused into the astrocyte syncytium, which will block not only synaptically induced astrocyte G_q_ GPCR Ca^2+^ elevations, but also resting astrocyte Ca^2+^ levels. Manipulation of resting astrocyte Ca^2+^ may affect neuronal activity *via* mechanisms including changes in neurotransmitter uptake or ambient neurotransmitter concentrations. Third, propagating intercellular Ca^2+^ waves between astrocytes indicating release of the gliotransmitter ATP have not been substantiated in intact brain tissue from healthy subjects, arguing against gliotransmitter release in normal physiology. Last, because the endogenous means by which synaptically induced astrocyte Ca^2+^ elevations occur is through activation of G_q_ GPCRs, the kinetics of astrocyte Ca^2+^ elevations seem too slow to modulate neuronal synaptic activity occurring on a millisecond timescale (Figure 4). It only seems possible that astrocytes could modulate neuronal activity in a G_q_ GPCR-dependent manner seconds after the initiating neuronal stimulus, supporting the concept that astrocytes act as “re-suppliers” to support synaptic transmission through recovery of neuronal metabolism or re-establishment of extracellular transmitter homeostasis. The fact that neuronally evoked G_q_ GPCR Ca^2+^ elevations in astrocytes are not sufficient to trigger gliotransmitter release to acutely modulate neurotransmission in the healthy brain, but instead would have a role in maintaining the homeostatic levels of ambient transmitters or ions, has profound implications in our understanding of synaptic transmission. Indeed, the concept of astrocytes being the third functional element of the synapse, in addition to the pre- and postsynaptic compartments, by releasing gliotransmitters to acutely (millisecond timescale) influence synaptic transmission affects the interpretation of a broad range of findings in neurophysiology. Thus, we propose that synaptically induced G_q_ GPCR Ca^2+^-dependent release of gliotransmitters (i.e., gliotransmission) be rebranded as gliomodulation.

Emerging research is suggesting that Ca^2+^-dependent release of gliotransmitters (glutamate and ATP) is also dependent on activated microglia, reactive astrocytes, and inflammatory molecules (Figure 5). These molecules include ATP, SDF-1α, TNFα, and PGE_2_, mediators that are rapidly upregulated and secreted by activated microglia and reactive astrocytes. This new information is exciting for the following reasons: First, it provides a possible explanation for the disparate findings among research groups regarding the Ca^2+^-dependence of gliotransmitter release from astrocytes (Agulhon et al., 2008; Hamilton and Attwell, 2010). Second, because it appears that activated microglia and increased release of inflammatory mediators and glutamate can occur rapidly, within seconds to minutes, glutamate release by reactive astrocytes may be a key early event in the progression of changes leading to neuronal hyperexcitability and excitotoxicity in neurological disorders and neuroinflammatory and neurodegenerative diseases. The observation that Ca^2+^-dependent release of glutamate by astrocytes depends on glial mediators, as well as the second-to-minute time course of this response, argues that gliomodulation but not gliotransmission is involved. Definitive proof for Ca^2+^-dependent astrocyte glutamate release to occur *via* exocytosis awaits further evidence, and mechanisms other than exocytosis might also be at work including release through volume-regulated anion channels, pore-forming P2X7 channels, connexin hemichannels, or reversal of glutamate uptake (Haskew-Layton et al., 2008; Malarkey and Parpura, 2008;Zhang et al., 2008, 2011; Li et al., 2012). It will be important in future studies assessing the impact of astrocyte transmitter release in pathological conditions to dissociate between these alternative release pathways. The IP_3_R2 KO mice will be particularly useful for dissociating astrocyte Ca^2+^ specifically from other astrocyte transmitter release mechanisms. Future experiments that can measure and manipulate secretion and levels of inflammatory mediators *vivo* may also provide more compelling information regarding the conditions required for inflammatory mediator/Ca^2+^-dependent release of gliotransmitters by astrocytes. Overall, these findings provide promising new glial targets for therapeutical intervention to treat a wide range of central nervous system pathologies.

## Conflict of Interest Statement

The authors declare that the research was conducted in the absence of any commercial or financial relationships that could be construed as a potential conflict of interest.
